# Adaptive Filtering: Issues, Challenges, and Best-Fit Solutions Using Particle Swarm Optimization Variants

**DOI:** 10.3390/s23187710

**Published:** 2023-09-06

**Authors:** Arooj Khan, Imran Shafi, Sajid Gul Khawaja, Isabel de la Torre Díez, Miguel Angel López Flores, Juan Castañedo Galvlán, Imran Ashraf

**Affiliations:** 1 College of Electrical and Mechanical Engineering, National University of Sciences and Technology (NUST), Islamabad 44000, Pakistan; arooj.khan399@gmail.com (A.K.); imranshafi@ceme.nust.edu.pk (I.S.); sajid.gul@ceme.nust.edu.pk (S.G.K.); 2 Department of Signal Theory and Communications and Telematic Engineering, University of Valladolid, Paseo de Belén 15, 47011 Valladolid, Spain; 3 Research Group on Foods, Universidad Europea del Atlántico, Isabel Torres 21, 39011 Santander, Spain; miguelangel.lopez@uneatlantico.es (M.A.L.F.); juan.castanedo@uneatlantico.es (J.C.G.); 4 Research Group on Foods, Universidad Internacional Iberoamericana, Campeche 24560, Mexico; 5 Instituto Politécnico Nacional, UPIICSA, Ciudad de Mexico 04510, Mexico; 6 Universidad Internacional Iberoamericana Arecibo, Arecibo, PR 00613, USA; 7 Department of Projects, Universidade Internacional do Cuanza, Cuito EN250, Bié, Angola; 8 Department of Information and Communication Engineering, Yeungnam University, Gyeongsan 38541, Republic of Korea

**Keywords:** adaptive filtering, particle swarm optimization, bit error rate, signal quality

## Abstract

Adaptive equalization is crucial in mitigating distortions and compensating for frequency response variations in communication systems. It aims to enhance signal quality by adjusting the characteristics of the received signal. Particle swarm optimization (PSO) algorithms have shown promise in optimizing the tap weights of the equalizer. However, there is a need to enhance the optimization capabilities of PSO further to improve the equalization performance. This paper provides a comprehensive study of the issues and challenges of adaptive filtering by comparing different variants of PSO and analyzing the performance by combining PSO with other optimization algorithms to achieve better convergence, accuracy, and adaptability. Traditional PSO algorithms often suffer from high computational complexity and slow convergence rates, limiting their effectiveness in solving complex optimization problems. To address these limitations, this paper proposes a set of techniques aimed at reducing the complexity and accelerating the convergence of PSO.

## 1. Introduction

Particle swarm optimization (PSO) is a computational optimization technique inspired by the collective behavior of swarms. It was originally proposed by Kennedy and Eberhart in 1995 [1] and has since become a popular and effective method for solving various optimization problems [2]. PSO simulates the social behavior of a swarm of particles, where each particle represents a potential solution in the search space [3]. The particles move through the search space, adjusting their positions based on their own experience and the experiences of their neighboring particles. The objective is to find the optimal solution by iteratively updating the positions of the particles in search of better solutions.

This study makes significant contributions to the field of adaptive equalization by exploring PSO techniques. Motivated by the need to enhance the optimization capabilities of PSO in communication systems [4], the research aimed to address the limitations of traditional PSO algorithms, such as slow convergence rates and high computational complexity [5]. The study investigated the combination of PSO with other optimization algorithms, adaptive mechanisms, multi-objective optimization, the constriction factor approach, and the dynamic neighborhood topology [6]. The primary research question driving this study is how to improve PSO for adaptive filters in terms of convergence, accuracy, and adaptability. By answering this question, the research provides valuable insights and recommendations to optimize the tap weights of adaptive filters, thereby enhancing signal quality and mitigating distortions in communication systems. The primary research questions driving this study are as follows:

- RQ1: How can the optimization capabilities of PSO be further enhanced for adaptive filters in the context of equalization?
- RQ2: How does the resemblance of PSO with algorithms such as the the least mean squares (LMS) and recursive least squares (RLS) contribute to the understanding and development of adaptive filters?
- RQ3: What are the recent advancements in PSO algorithms, such as ring topology, dynamic multi-swarm PSO, and fully informed PSO, and how do they improve the performance of adaptive filtering?
- RQ4: How does the dynamic neighborhood concept in PSO contribute to better exploration and exploitation of the search space?
- RQ5: What are the benefits and challenges of hybridization techniques, such as hybrid PSO and cooperative PSO, in improving the optimization capabilities of PSO?
- RQ6: What are the time and space complexity considerations of PSO algorithms, and how do they impact the scalability and efficiency of the optimization process?
- RQ7: What are the limitations and challenges of PSO to achieve a better convergence rate?

The study sought to explore and propose various techniques to improve the convergence, accuracy, and adaptability of PSO algorithms. The research investigated the combination of PSO with other optimization algorithms, the introduction of adaptive mechanisms, the application of multi-objective optimization, and the utilization of the constriction factor approach and dynamic neighborhood topology. By answering these research questions, the study aimed to provide insights and recommendations for optimizing the tap weights of adaptive filters using PSO in communication systems.

This review is further divided into six parts. Section 2 elaborates on the techniques used for adaptive equalization. PSO, its time complexity, and its resemblance to other optimization algorithms are discussed in Section 3. Section 4 provides a comprehensive overview of PSO approaches used for adaptive filtering including the comparative analysis of PSO variants. Hybrid PSO, the best-fit PSO solution for adaptive filtering, is discussed in Section 5 concerning its advantages and disadvantages. In the end, the conclusion and future directions are given in Section 6.

Section 2 is dedicated to addressing RQ1 and RQ2. The answers to RQ3 and RQ4 can be found in Section 3. Section 4 presents the discussions regarding RQ5. Lastly, Section 5 delves into the responses to RQ6 and RQ7.

## 2. Techniques Used for Adaptive Equalization

Adaptive equalization is a fundamental signal-processing technique utilized in numerous communication systems to improve the quality and reliability of transmitted data [7]. It serves as a crucial step in combating the detrimental effects of channel impairments, such as multipath propagation and frequency response variations, which can introduce inter-symbol interference (ISI) and degrade the received signal quality [8,9]. The primary goal of adaptive equalization is to dynamically adjust the characteristics of the received signal to closely align with the desired signal [10], effectively mitigating distortions and restoring the fidelity of the transmitted data [11]. To achieve adaptive equalization, a diverse range of techniques has been developed [12], each with its own approach and advantages. These techniques are designed to adaptively modify the parameters or coefficients of the equalizer based on the characteristics of the channel and the received signal. By continuously monitoring and updating the equalizer, it can adapt to the changing conditions of the communication channel and optimize its performance accordingly.

The field of adaptive equalization has witnessed significant advancements and innovation over the years [13], driven by the increasing demands for high-speed data transmission and reliable communication systems [14,15]. Researchers and engineers have explored various approaches including algorithmic optimization techniques, machine learning algorithms, and advanced signal-processing methods, to enhance the performance of adaptive equalization [16,17]. These techniques aim to strike a balance between computational complexity, convergence speed, and adaptability to different channel conditions, providing robust and efficient solutions for adaptive equalization in a wide range of applications [18]. The choice of adaptive equalization technique depends on several factors, such as the specific characteristics of the channel [19], the desired performance metrics [20], the available computational resources, and the trade-off between complexity and effectiveness [21]. As the field continues to evolve, researchers are constantly pushing the boundaries of adaptive equalization techniques, seeking novel approaches to address the challenges posed by emerging communication technologies and ever-changing channel conditions. By harnessing the power of adaptive equalization, communication systems can achieve higher data rates, improved spectral efficiency, and enhanced reliability, paving the way for seamless and efficient transmission of information in diverse environments. Adaptive equalization can be achieved using various techniques [22], each with its unique approach and advantages. Some of the techniques include LMS, RLS, PSO, genetic algorithms (GAs), and deep learning, which are discussed below.

### 2.1. Least-Mean-Squared Error

LMS is an adaptive filtering algorithm widely used for adaptive equalization [23]. It aims to minimize the mean squared error between the desired signal and the filter output [24]. LMS updates the filter coefficients iteratively based on the instantaneous estimation error and the input signal [25,26,27]. In the context of adaptive equalization, LMS is employed to adjust the equalizer’s coefficients and compensate for distortions caused by the channel [28]. By continuously adapting the filter coefficients, LMS enables the equalizer to adapt to changing channel conditions and optimize its performance [29]. LMS is known for its simplicity and ease of implementation, making it a popular choice in various communication systems.

### 2.2. Recursive Least Squares

RLS is another popular adaptive filtering algorithm used for adaptive equalization [30]. It recursively updates the filter coefficients based on the instantaneous estimation error and the input signal [31]. RLS utilizes a matrix inversion technique to achieve optimal filter updates [32]. In the context of adaptive equalization, RLS offers fast convergence and provides accurate filter estimation [33]. However, RLS has higher computational complexity and memory requirements compared to LMS [34]. Despite these limitations, RLS is preferred in applications that require rapid convergence and optimal filter updates [35].

### 2.3. Particle Swarm Optimization

PSO is a population-based stochastic optimization algorithm inspired by social behavior [36]. In the context of adaptive equalization, PSO is utilized to optimize the equalizer’s coefficients by iteratively exploring a multidimensional search space [37]. PSO works by simulating the movement of particles, where each particle represents a potential solution. By leveraging the best experiences of the swarm and their own experiences, particles dynamically adjust their positions in the search space to find optimal solutions [38]. PSO provides a global search capability, allowing it to handle complex and nonlinear optimization problems [39]. This makes PSO suitable for adaptive equalization tasks that require optimal filter coefficients and enhanced convergence [39]. The detailed analysis of the PSO algorithm and its variants for adaptive equalization is discussed in later sections.

### 2.4. Genetic Algorithms

The GA is an optimization technique inspired by the process of natural selection and genetics [40]. In the context of adaptive equalization, the GA is employed to evolve a population of candidate solutions towards the optimal solution [41]. The GA involves the use of selection, crossover, and mutation operators to iteratively improve the quality of solutions [42]. The GA can handle complex optimization problems and provides a diverse set of solutions [43]. By using appropriate genetic operators and fitness evaluation criteria, the GA can effectively optimize the equalizer’s coefficients for adaptive equalization.

### 2.5. Deep Learning

Deep learning techniques, specifically deep neural networks, are increasingly used for adaptive equalization tasks [44]. Deep learning approaches involve training neural networks to learn the mapping between the received signal and the desired signal [45]. In the context of adaptive equalization, deep neural networks can model the complex and nonlinear relationship between the input signal and the equalized output [46]. By utilizing large amounts of training data and employing sophisticated network architectures, deep learning techniques can adapt to a wide range of channel characteristics and achieve superior equalization performance [47,48]. A deep learning approach requires significant computational resources, substantial training data, and careful regularization techniques to mitigate overfitting [49]. Table 1 provides a comprehensive overview of the pros and cons of techniques used for adaptive equalization.

## 3. Particle Swarm Optimization

### 3.1. Standard PSO Algorithm

PSO is an optimization method inspired by swarm behavior observed in nature, where a population of particles represents the optimization parameters [53]. These particles collectively search for optimal solutions within a multi-dimensional search space. The objective of the algorithm is to converge toward the best-possible values for each parameter [54]. The fitness of each particle is evaluated using a fitness function, which quantifies the quality of the particle’s solution estimate [5,55]. Each particle maintains two state variables: its position (x(i)) and velocity (v(i)), where *i* represents the iteration index. The fitness of a particle is determined by evaluating a cost function associated with its solution estimate. Through information sharing, each particle combines its own best solution with the best solution found by the entire swarm, adjusting its search pattern accordingly. This iterative process continues until an optimal solution is reached or a termination criterion is met. The equation of the standard PSO algorithm is given as

(1) $$v_{k}^{d}\left(i+1\right)=v_{k}^{d}\left(i\right)+c_{1}.r_{1},k\left(i\right).\left(p_{k}^{d}-x_{k}^{d}\left(i\right)\right)+c_{2}.r_{2},k\left(i\right).\left(g^{d}-x_{k}^{d}\left(i\right)\right)$$

(2) $$x_{k}^{d}\left(i+1\right)=x_{k}^{d}\left(i\right)+v_{k}^{d}\left(i+1\right)$$

The equation represents the velocity and position update mechanism in PSO. The velocity is updated by combining the particle’s previous velocity, the cognitive component based on its personal best solution, and the social component based on the global best solution found by the swarm. This combination allows the particle to maintain its momentum, explore its individual best solution, and be influenced by the overall best solution. The updated velocity is then used to update the particle’s position, determining its next location in the search space. The updated version of PSO having an inertia term is given below:

(3) $$v_{k}^{d}\left(i+1\right)=v_{k}^{d}\left(i\right).r_{1},k\left(i\right).\left(p_{k}^{d}-x_{k}^{d}\left(i\right)\right)+c_{2}.r_{2},k\left(i\right).\left(g^{d}-x_{k}^{d}\left(i\right)\right)$$

where $c_{1}$ represents the cognitive term, $c_{2}$ represents the social term, *d* is the dimension of the particles, $P_{k}$ is the local best, *g* is the global best of the particle, and $r_{1}$ and $r_{2}$ are the random variables, their range lying between 0 and 1 [56], while the momentum of a particle is controlled by inertia, represented by *w*.

**Figure 1:**
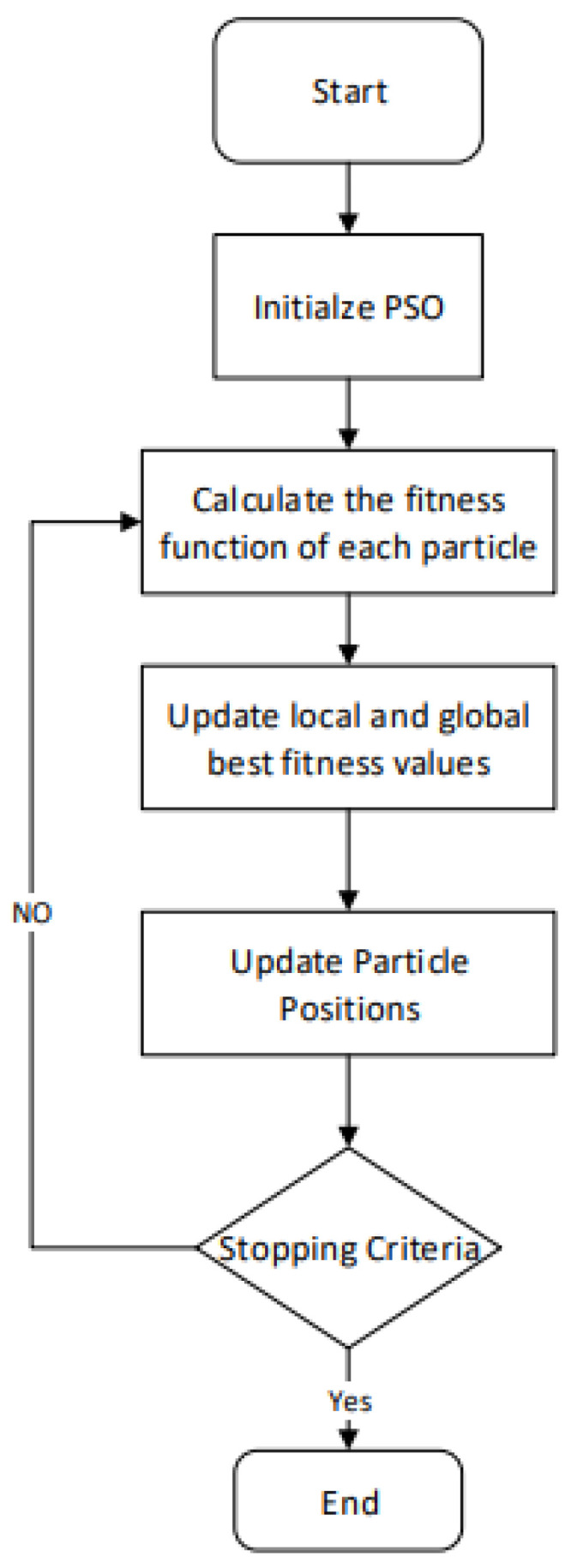
Flow chart of standard PSO algorithm.

When the inertia of the particle is zero, the model will only explore and become independent of past values. The convergence rate of the PSO algorithm refers to the speed at which the algorithm converges toward an optimal solution [57]. The convergence rate of PSO can be influenced by various factors, including problem complexity, population size, inertia weight, acceleration coefficients, and termination conditions [58]. The flow chart of the standard PSO algorithm mentioned in [59] is shown in Figure 1.

**Figure 2:**
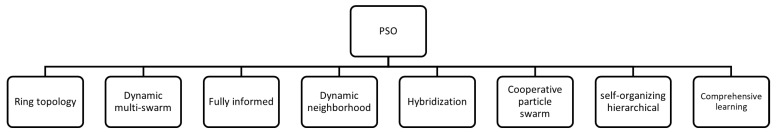
Variants of particle swarm optimization.

PSO has the potential for fast convergence due to its ability to share information among particles in the swarm [60]. Collective knowledge sharing enables particles to converge towards promising regions of the search space [61]. However, the convergence rate of standard PSO can be affected by the balance between exploration and exploitation. If the exploration is too dominant, the algorithm may take longer to converge. On the other hand, if the exploitation is too dominant, the algorithm may converge prematurely to local optima [62]. To enhance the convergence rate, several strategies can be employed. One approach is to adaptively adjust the parameters of the algorithm during the optimization process. This includes modifying the inertia weight and acceleration coefficients to balance exploration and exploitation at different stages of the optimization [63]. Different variants of PSO algorithms exist in the literature, shown in Figure 2, to achieve better complexity and faster convergence.

### 3.2. Resemblance of Artificial Intelligence and PSO

PSO and artificial intelligence (AI) are two distinct computational approaches with both similarities and differences [64]. Both PSO and AI share the common goal of solving complex problems and optimizing system performance. They rely on algorithms and techniques to learn from data, make decisions, and improve overall performance. Furthermore, both PSO and AI have versatile applications across various domains, including optimization, pattern recognition, decision-making, and control systems. There are notable differences between PSO and AI. PSO is a specific optimization algorithm inspired by the collective behavior of bird flocks or fish schools [65]. It is a population-based metaheuristic algorithm that iteratively adjusts the positions of particles in search of the optimal solution. On the other hand, AI is a broader field encompassing various techniques, including but not limited to PSO, such as neural networks, genetic algorithms, and expert systems [66]. While PSO is primarily designed for optimization problems and focuses on finding the best solution within a given search space, AI encompasses a wider range of techniques. These techniques can include machine learning, natural language processing, robotics, and more. AI techniques can be applied to various problem domains [67], not necessarily limited to optimization. PSO operates based on the principles of collective intelligence and social behavior, where particles communicate and learn from each other to find the best solution. In contrast, AI approaches can involve learning from data, simulating human cognitive processes, or mimicking intelligent behavior using different algorithms and methodologies.

### 3.3. Resemblance with Least Mean Square and Recursive Least Squares

The PSO, LMS, and RLS algorithms share certain resemblances in terms of their learning mechanisms and optimization objectives. PSO is an algorithm where particles within a swarm collectively explore and exploit the search space to find optimal solutions. Similarly, both the LMS and RLS algorithms are adaptive filtering techniques used in signal processing and parameter estimation [68]. They aim to adjust the internal parameters iteratively to minimize the error between the predicted and actual outputs.

One resemblance between PSO, LMS, and RLS is their learning mechanism. In PSO, particles adjust their positions and velocities based on their individual experiences and the collective knowledge of the swarm [69]. This learning process allows particles to explore the search space and exploit promising regions. Similarly, in LMS and RLS, the algorithms update their weight vectors or coefficients based on the input data and the discrepancy between the predicted and actual outputs. This iterative learning mechanism in all three algorithms enables them to converge toward optimal solutions or parameter estimates.

### 3.4. Applications of PSO

Due to advancements in and modifications of PSO, various applications have been found in the literature [70] due to its ability to efficiently search for optimal solutions. PSO can be applied to optimize mathematical functions with multiple variables. By exploring the search space, particles can locate the global minimum or maximum of a function. This application is particularly useful in fields such as engineering design, data analysis, and financial modeling.

In image and signal processing, PSO has been employed for image and signal processing tasks [71]. It can optimize parameters in image reconstruction, denoising, feature extraction, and object recognition. PSO algorithms have shown promising results in optimizing parameters for image- and signal-processing techniques, enhancing the quality and efficiency of these processes [72].

PSO can be used to train the weights and biases of neural networks. It has been employed as an alternative to traditional optimization algorithms, such as backpropagation, to improve the training process and avoid local optima. PSO-based training algorithms can enhance the convergence speed and accuracy of neural networks, making them more effective in pattern recognition, classification, and prediction tasks. They are also used to solve optimization problems in power systems [70]. They can optimize various aspects such as power flow, unit commitment, economic dispatch, and capacitor placement. PSO-based approaches enable efficient utilization of power resources, leading to improved power system operation, reduced costs, and enhanced stability. They can be used for feature selection in machine learning and data-mining tasks. By selecting a subset of relevant features, PSO helps in dimensionality reduction, improving classification accuracy and reducing computational complexity. This application is particularly useful in areas such as text mining, bioinformatics, and image recognition [73].

PSO is also used to optimize vehicle-routing problems, including route planning, delivery scheduling, and fleet management. By considering factors such as distance, capacity, and time constraints, PSO algorithms can determine efficient routes and schedules, minimizing transportation costs and improving logistics operations. Another application of PSO in electronics is in the optimization of antenna design [74]. Antennas are crucial components in wireless communication systems, and their performance greatly impacts signal reception and transmission. Moreover, in radar waveform design, PSO can optimize the characteristics of radar waveforms, such as pulse duration, modulation schemes, and frequency characteristics, to enhance target detection, resolution, and interference mitigation. By iteratively adjusting particle positions representing waveform parameters, PSO can efficiently explore the design space and converge on optimal solutions that maximize radar performance. This enables radar systems to improve their capabilities in detecting and tracking targets, reducing interference, and enhancing overall operational efficiency.

### 3.5. Time and Space Complexity of PSO

**Figure 3:**
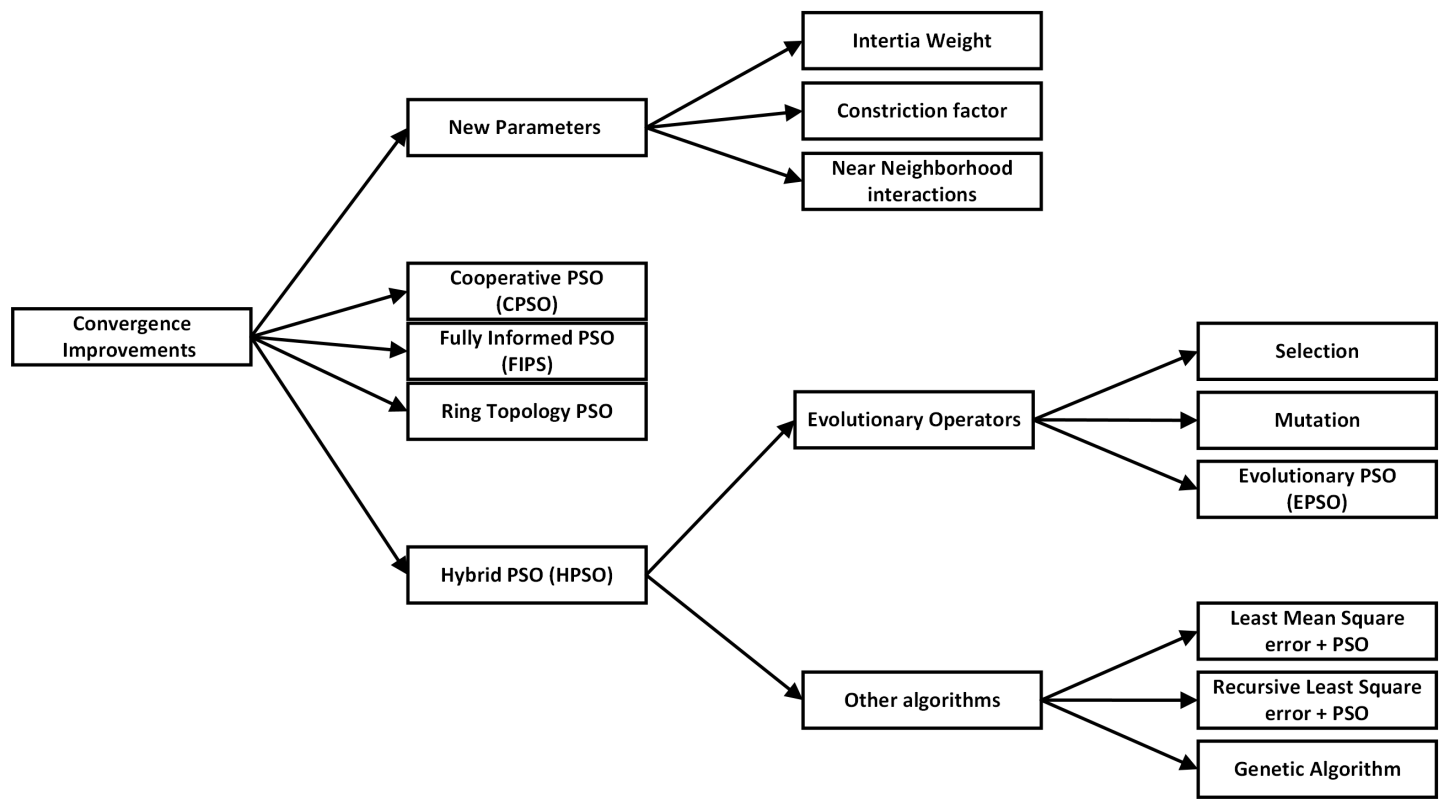
Convergence improvements of different variants of PSO.

In terms of time complexity, the main computational cost of PSO lies in evaluating the objective function for each particle in each iteration. The objective function represents the problem to be optimized and can vary in complexity depending on the problem domain. Therefore, the time complexity of PSO is closely related to the evaluation time of the objective function [75]. In each iteration, all particles need to evaluate their positions, update their personal bests and the global best, and adjust their velocities and positions. This process continues until a termination condition is met. The number of iterations required for convergence depends on various factors such as the problem complexity, the size of the search space, and the convergence speed of the swarm [76]. Generally, the time complexity of PSO is considered to be moderate, as it typically requires a reasonable number of iterations to converge to an acceptable solution [77]. The greater number of iterations leads to the requirement of large memory. PSO requires memory to store the positions, velocities, personal bests, and global best of each particle in the swarm [78]. The amount of memory required is proportional to the population size, which is typically determined by the problem being solved. PSO may also require memory to store auxiliary variables, such as acceleration coefficients and parameters controlling the swarm behavior. The space complexity of PSO is, therefore, determined by the memory requirements for storing the swarm’s state and other relevant variables. The space complexity is generally considered to be reasonable, as it scales linearly with the population size and does not depend on the size of the search space. Figure 3 shows the improvements in PSO over time to quickly converge to the optimal solution.

### 3.6. Recent Advancements in PSO for a Better Convergence Rate

In recent years, significant advancements have been made for PSO, enhancing its performance and expanding its applications [73]. These advancements have focused on addressing various challenges and improving the algorithm’s effectiveness. One notable advancement is the development of techniques to handle large-scale optimization problems [79]. Researchers have devised parallel and distributed PSO algorithms, which utilize multiple computing resources to tackle computationally intensive tasks efficiently [80]. This advancement has opened the door to optimizing complex problems that were previously unfeasible with traditional PSO approaches. Another noteworthy development is the integration of PSO with machine learning techniques [81]. By combining PSO with algorithms such as neural networks or deep learning models, the optimization process becomes more robust, enabling the solution of intricate problems and improving prediction tasks. Additionally, self-adaptive PSO algorithms have emerged, allowing for dynamic adjustments of algorithm parameters during optimization. These algorithms utilize adaptive mechanisms to fine-tune parameters based on the particles’ performance, leading to improved convergence and solution quality. These advancements in PSO continue to push the boundaries of optimization capabilities, making it a valuable tool for tackling real-world challenges. A brief overview of the advancements in the PSO algorithm is presented in Table 2. Over time, different variants of the PSO algorithm have been introduced, which are discussed in the subsequent sections.

#### 3.6.1. Ring Topology in Particle Swarm Optimization

**Figure 4:**
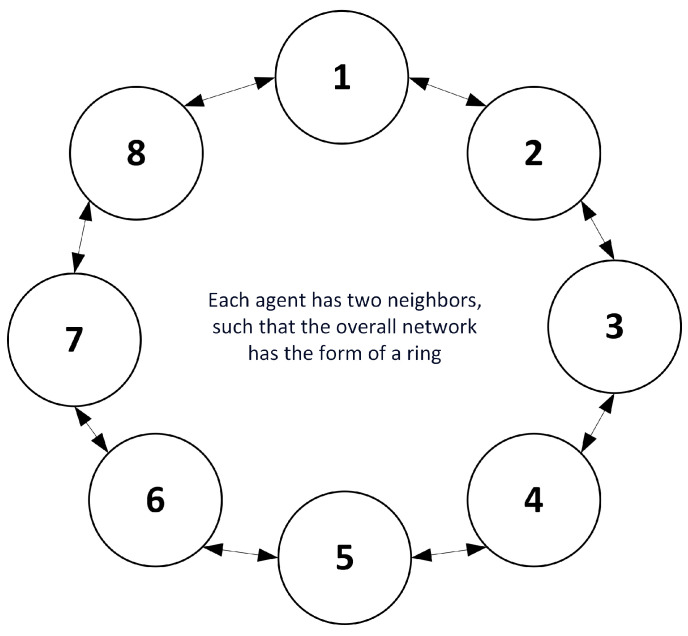
Ring topology PSO algorithm.

The ring topology in PSO is a variation of the algorithm where the particles are arranged in a circular ring structure instead of a fully connected network [99]. Figure 4 shows the settings of the ring topology in PSO. In this topology, each particle is only connected to its immediate neighbors, creating a cyclic structure [100]. In the ring topology, the communication and information sharing among particles are limited to the adjacent neighbors. This arrangement allows for a more-localized interaction, as each particle only exchanges information with its neighbors, rather than the entire swarm [101]. The neighbor particles influence the velocity and position updates of a given particle. During each iteration, the particle evaluates its fitness based on the objective function and updates its personal best position. It then exchanges information with its neighbors, considering both its own best position and the best position found by its neighbors. The particle’s velocity and position are updated using the information obtained from these local interactions [102]. In the ring topology, the updating equation for velocity and position remains the same as in the standard PSO algorithm. However, the difference lies in the information-sharing process, where particles only consider the personal best position and the best position of their adjacent neighbors when updating their velocity and position [103].

#### 3.6.2. Dynamic Multi-Swarm Particle Swarm Optimization

**Figure 5:**
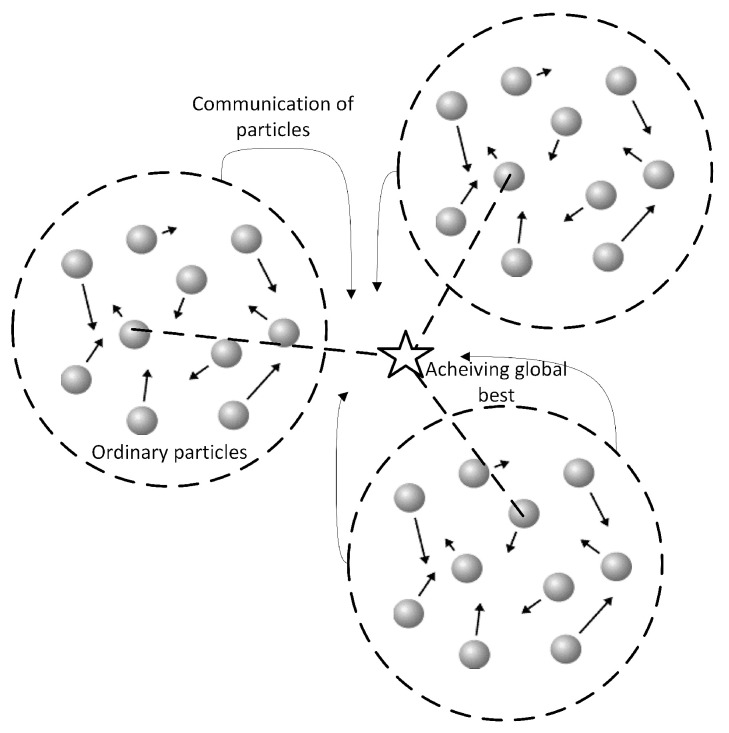
Dynamic multi-swarm PSO.

Dynamic multi-swarm PSO is an extension of the standard PSO algorithm that incorporates the concept of multiple swarms to improve optimization performance in dynamic environments [104,105]. Unlike the standard PSO, where all particles belong to a single swarm, dynamic multi-swarm PSO divides the population into multiple subgroups or swarms, each with its own characteristics and behavior [106], as shown in Figure 5. In this approach, each swarm operates independently, with its own set of particles exploring the search space. The swarms can be formed based on different criteria, such as spatial division or clustering techniques [107]. Each swarm maintains its own local best positions (pbest) and global best position (gbest), representing the best solutions found within the swarm and the overall best solution obtained among all swarms, respectively. The use of multiple swarms in dynamic multi-swarm PSO provides several advantages [108]. It allows for a more-distributed exploration of the search space, enabling the algorithm to better handle dynamic changes and avoid being trapped in local optima [109]. The dynamic reconfiguration of swarms facilitates adaptation to changes and improves the algorithm’s robustness and responsiveness.

#### 3.6.3. Fully Informed Particle Swarm Optimization

Fully informed particle swarm optimization (FIPS) enhances the communication and information exchange among particles within a swarm [110]. In FIPS, each particle not only considers its personal best solution (pbest) and the global best solution (gbest), but also incorporates information from the best solutions of its neighboring particles. This fully informed approach allows for a more-comprehensive exploration of the search space and can lead to improved optimization performance. The communication topology can take different forms, such as a fully connected network or a spatially defined neighborhood structure [111]. Each particle maintains a set of information about its neighbors’ best solutions, known as the “flock” information. This flock information consists of the position and fitness values of the neighboring particles’ best solutions. The fully informed communication mechanism in FIPS facilitates cooperative interactions among particles, enabling them to share valuable information and guide each other toward promising regions in the search space [112]. This enhanced communication promotes a balance between exploration and exploitation, helping to avoid premature convergence to sub-optimal solutions.

#### 3.6.4. Dynamic Neighborhood in Particle Swarm Optimization

The dynamic neighborhood in PSO is an adaptive mechanism that allows particles to adjust their communication network or neighborhood structure during the optimization process [113]. Unlike the traditional fixed neighborhood approach, where each particle interacts with a fixed set of neighbors, the dynamic neighborhood enables particles to dynamically form and update their local neighborhoods based on the problem dynamics or specific optimization requirements [114]. The neighborhood structure is not predetermined, but evolves over time. Initially, particles are assigned to random neighborhoods or a predefined initial configuration [115]. As optimization progresses, particles continuously evaluate their performance and exchange information with their neighbors. Based on this information, particles may reconfigure their neighborhoods by adding or removing neighboring particles, thereby dynamically adjusting the communication network [116].

#### 3.6.5. Hybridization in Particle Swarm Optimization

**Figure 6:**
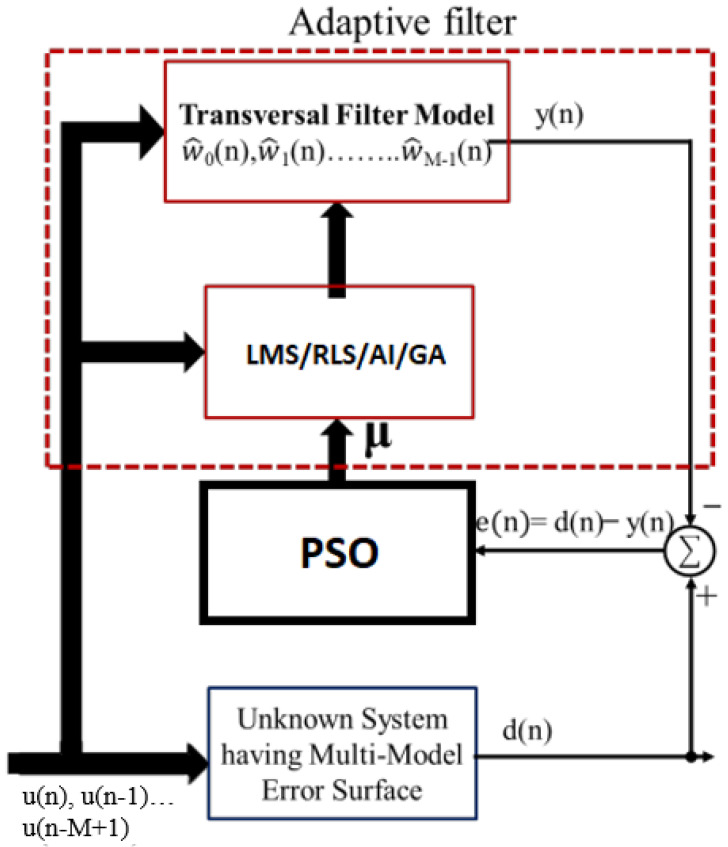
Flow chart of hybrid PSO algorithm.

Hybridization in PSO (HPSO) refers to the integration of PSO with other optimization techniques or problem-specific heuristics to enhance its performance and overcome its limitations [117,118]. By combining the strengths of multiple algorithms, HPSO aims to achieve improved exploration and exploitation capabilities, increased convergence speed, and better overall solution quality [119]. One common approach is to combine PSO with local search methods, such as gradient descent or hill climbing, to refine the solutions obtained by PSO [120]. This combination allows for a more-thorough exploration of the search space and can help escape local optima. HPSO can be customized based on the specific characteristics of the problem domain. Problem-specific heuristics or knowledge can be incorporated to guide the search process. The flow chart of the HPSO algorithm for adaptive equalization is shown in Figure 6.

#### 3.6.6. Cooperative Particle Swarm Optimization

**Figure 7:**
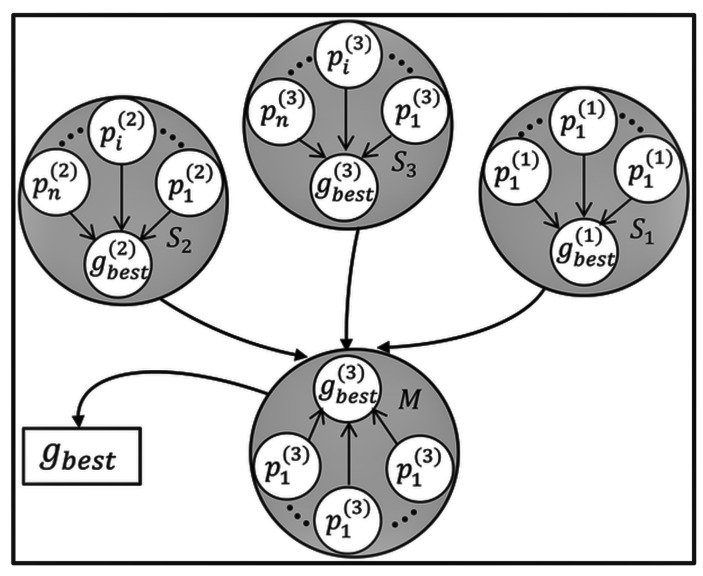
Flow chart of the cooperative PSO algorithm.

Cooperative PSO (CPSO) is an extension of the standard PSO algorithm that promotes collaboration and information sharing among multiple sub-swarms or groups of particles [121]. Figure 7 shows the graphical presentation of CPSO working. In cooperative PSO, instead of having a single global best solution for the entire swarm, each sub-swarm maintains its own local best solution, and particles from different sub-swarms communicate and cooperate to collectively search for optimal solutions [122]. The sub-swarms operate independently, exploring different regions of the search space. Periodically, particles exchange information about their best solutions with particles from other sub-swarms [123]. This information sharing allows particles to gain insights from successful regions discovered by other sub-swarms, promoting exploration beyond local optima and facilitating a more-thorough exploration of the search space.

#### 3.6.7. Self-Organizing Hierarchical Particle Swarm Optimization

In this approach, particles are organized into a hierarchical structure, where higher-level particles oversee the behavior of lower-level particles [124]. This hierarchical arrangement enables a more-efficient search process by coordinating the exploration of different regions of the search space. It is an advanced variant of the standard PSO algorithm, which introduces a hierarchical organization among particles to enhance their exploration and exploitation capabilities [125]. The higher-level particles guide the lower-level particles based on their own experiences and the information they receive from the lower-level particles [126]. The higher-level particles take on a supervisory role, adjusting the influence of different components in the PSO equation to balance global and local search tendencies [127]. They communicate with and provide guidance to the lower-level particles, influencing their search patterns and facilitating the discovery of promising regions. This allows for a distributed and coordinated exploration, as different levels of particles focus on different regions and scales within the search space [128,129]. The hierarchical organization helps to mitigate the risk of premature convergence and aids in escaping local optima by facilitating exploration in unexplored regions.

#### 3.6.8. Comprehensive Learning Particle Swarm Optimization

Comprehensive learning PSO (CLPSO) is an advanced variant of the standard PSO algorithm that introduces a comprehensive learning strategy [130]. In CLPSO, particles not only learn from their personal best and global best solutions, but also learn from other randomly selected particles within the swarm. This comprehensive learning mechanism allows for a broader exploration of the search space and promotes the exchange of valuable information among particles. Incorporating knowledge from multiple sources enhances the diversity of search trajectories, facilitates the discovery of new regions, and improves the convergence speed and solution quality [131]. The comprehensive learning strategy in CLPSO enables particles to make more-informed decisions during the optimization process, leveraging the collective intelligence of the swarm to achieve better performance in solving complex optimization problems.

## 4. PSO Techniques for Adaptive Equalization

PSO variants have been extensively utilized to enhance adaptive equalization in communication systems. These variants aim to improve the optimization capabilities of PSO algorithms and enable better performance of adaptive filters [118]. Dynamic multi-swarm PSO introduces multiple swarms dynamically adapting to different regions of the search space [132]. By assigning specific areas of exploration to each swarm, this variant efficiently explores and exploits the equalizer’s tap weight space, making it suitable for handling complex frequency response variations and mitigating distortions [133]. Another variant, FIPS, enhances information exchange among particles by allowing them to communicate with all others in the swarm. This global information sharing promotes better exploration of the search space, leading to improved convergence and accuracy in adaptive equalization tasks [133].

HPSO combines PSO with other optimization algorithms such as the GA or simulated annealing (SA) [134]. This integration enables leveraging the strengths of different algorithms [135], allowing efficient navigation of the tap weight space and achieving improved convergence and adaptation in the presence of frequency response variations [136]. Cooperative PSO introduces cooperation mechanisms among particles, facilitating information exchange and adaptation based on shared knowledge. In adaptive equalization, cooperative PSO enhances exploration and adaptation capabilities, particularly when dealing with varying channel conditions [137]. Self-organizing hierarchical PSO introduces a hierarchical structure among particles, promoting effective exploration and exploitation within localized regions of the search space. This variant adapts to different subsets of the tap weight space, enhancing adaptability to varying channel conditions and improving equalization performance. Lastly, CLPSO incorporates a learning mechanism where particles adapt their behavior based on their own experiences and the best experiences of the swarm. By combining personal and swarm knowledge, CLPSO achieves faster convergence, improved exploitation of promising solutions, and better adaptation to frequency response variations in adaptive equalization tasks [138]. These PSO variants, with their unique characteristics and capabilities, have been successfully applied in adaptive equalization to optimize tap weights and enhance convergence rates, accuracy, and adaptability, ultimately improving the overall signal quality in communication systems [139].

### 4.1. Comparative Study of PSO Variants for Adaptive Filtering

PSO offers various enhancements and variations that can be applied to improve its performance and applicability in different domains. The ring topology in PSO enables particles to communicate and share information with their immediate neighbors, facilitating local exploration and exploitation. Dynamic multi-swarm PSO divides the population into multiple swarms that adapt dynamically to explore different regions of the search space, achieving a balance between exploration and exploitation. Fully informed PSO enhances the global exploration capability by allowing particles to have knowledge of the best solution found by their neighbors. Dynamic neighborhood PSO allows particles to change their set of neighbors dynamically, improving adaptability to changing problem conditions. The hybridization of PSO with other optimization techniques or problem-solving methods combines the strengths of different algorithms, leading to robust optimization in complex problem domains. Cooperative particle swarm PSO involves multiple swarms working together, facilitating knowledge exchange and cooperative behavior to tackle large-scale optimization problems. Self-organizing hierarchical PSO organizes particles in a hierarchical structure, allowing for efficient exploration and coordination across different levels. Comprehensive learning in PSO incorporates additional learning mechanisms or problem-specific knowledge, enhancing the algorithm’s efficiency and convergence toward optimal solutions. These enhancements and variations in PSO broaden its capabilities and make it applicable to a wide range of optimization problems across diverse domains. HPSO combines the strengths of PSO with other optimization techniques or problem-solving methods, making it a powerful and versatile approach for solving complex optimization problems. While it is not accurate to claim that hybrid PSO is universally better than all other optimization methods, it offers several advantages that make it highly effective in many scenarios. A critical summary of the advantages and limitations of PSO variants is provided in Table 3.

### 4.2. Performance Analysis

The selected PSO variants were utilized to analyze their efficacy for adaptive filtering in this study. The bit error rate (BER) is a measure of the error rate in a digital communication channel. It quantifies the probability of bit errors occurring during transmission. A lower BER indicates better channel performance, as it signifies fewer errors in the received bits. Factors that affect the BER include noise, interference, the modulation scheme, the coding techniques, and the channel characteristics. By analyzing and optimizing the BER, engineers can improve the overall reliability and quality of digital communication systems. A convergence analysis was conducted in an experimental setting, simulating a digital communication channel model. Six key parameters are defined to analyze the convergence of hybrid PSO. Population size *n*, data window size *N*, acceleration parameters c1 and c2, maximum velocity range, and the number of taps for the adaptive channel equalizer were selected. This methodical approach ensures the algorithm’s effectiveness and adaptability for various scenarios. To observe the behavior of HPSO, a convergence analysis was conducted in an experimental setting simulating a digital communication channel model. The analysis, as depicted in Table 4, demonstrated the stabilization of the convergence rate as the number of iterations (N) increased. This observation underscores HPSO’s capability to steadily refine its optimization process, an attribute essential for optimizing digital communication systems. The analysis of the convergence of HPSO over *N* iterations is shown in Table 4, which shows that the convergence rate becomes stabilized when *N* is large.

The performance of least mean squares (LMS) degrades compared to hybrid PSO due to its limited ability to handle nonlinear and non-convex optimization problems. Hybrid PSO incorporates global search capabilities and adaptive techniques, providing better convergence and optimization results. In the conducted experiment, the performance of three different optimization techniques LMS, the PSO VCF, and HPSO was evaluated in the context of a digital communication channel. The experiment involved varying SNR levels to simulate different channel conditions. The SNR represents the ratio of signal power to noise power and serves as a key factor in determining the quality of communication in noisy environments. For each SNR level, the BER was measured using the three optimization techniques: LMS, the PSO VCF, and HPSO. Table 5 shows the comparison of LMS, the PSO variable constriction factor (VCF), and HPSO. A lower BER signifies better channel performance, indicating fewer errors in received bits. The comparison of the BER values among the three techniques at different SNR levels provides insights into their respective abilities to mitigate errors and enhance communication quality.

The experiment revolved around evaluating four distinct PSO variants using the sphere function. The sphere function, a common optimization benchmark, calculates the sum of squared differences between the candidate solution and the optimal solution. The aim was to gauge the performance of these PSO variants in terms of mean function values across different dimensions, i.e., 30 and 60. Lower mean function values signify more-proficient optimization, thereby enabling a comparative analysis of the PSO techniques’ effectiveness in searching for optimal solutions. The experimental results demonstrated the superior performance of hybrid PSO compared to other variants of PSO in various optimization tasks. In a comparative study, different PSO variants, including standard PSO, adaptive PSO, and HPSO, were evaluated for their convergence speed and solution quality. The results revealed that HPSO outperformed the other variants in terms of both convergence speed and solution quality. HPSO demonstrated faster convergence, reaching the optimal or near-optimal solution more quickly compared to standard PSO and adaptive PSO. This was attributed to HPSO’s ability to balance exploration and exploitation through the combination of particle interactions and adaptive parameters. The solution quality achieved by HPSO was consistently superior to other variants. The algorithm’s hybrid nature, incorporating elements of both particle swarm optimization and local search techniques, allowed for better exploration of the search space, leading to improved solutions. HPSO effectively balanced global exploration to escape local optima with local exploitation to refine solutions, resulting in enhanced overall performance. Table 6 shows the results of 30D particle convergence.

Table 7 shows the convergence performance using 60D particles. The findings suggested that HPSO is a robust and effective optimization algorithm, which can outperform other PSO variants in various applications. Its ability to strike a balance between exploration and exploitation, along with the integration of local search techniques provide HPSO a competitive advantage. The superior performance of HPSO makes it a promising choice for optimization tasks where fast convergence and high-quality solutions are desired, such as channel adaptive equalization in communication systems, where accurate estimation and compensation of channel distortion are crucial for reliable data transmission. The evaluated dimensions ranged from 10 to 100. Lower mean function values indicate better optimization performance. The experiment involved applying these PSO variants to the sphere function, a well-known optimization benchmark. The sphere function computes the sum of squared differences between a candidate solution and the optimal solution. This experiment aimed to compare the effectiveness of the PSO techniques in achieving optimal solutions within different dimensional spaces.

Table 8 shows the mean function value (MFV) of different PSO algorithms. The simulation results consistently demonstrated the robustness and effectiveness of HPSO across various domains and problem types. Whether applied to engineering design optimization, function optimization, or other complex tasks, HPSO consistently outperformed the competing algorithms. These findings establish HPSO as a promising optimization approach that can provide significant benefits in terms of convergence speed and solution quality, making it an attractive choice for numerous real-world optimization problems.

The simulation results consistently demonstrated the robustness and effectiveness of HPSO across various domains and problem types. Whether applied to engineering design optimization, function optimization, or other complex tasks, HPSO consistently outperformed the competing algorithms. Table 9 and Table 10 present the MSE values under different signal-to-noise ratio (SNR) levels, which indicate the quality of the channel’s output signal. Lower MSE values signify better performance, indicating a closer approximation to the desired output. A negative MSE value can be an artifact of data representation or computation.

Comparing the techniques, it is evident that, under the given SNR conditions, the PSO-CCF, PSO-VCF, and HPSO methods consistently outperformed the basic LMS method, showcasing their efficacy in optimizing the adaptive filtering process for a linear channel. Among these three advanced methods, HPSO tended to yield the lowest MSE, suggesting its potential to provide the best approximation to the desired signal under different SNR scenarios. These findings establish HPSO as a promising optimization approach that can provide significant benefits in terms of convergence speed and solution quality, making it an attractive choice for numerous real-world optimization problems. The experiment was conducted for a linear time-invariant (LTI) system and nonlinear digital channel model having a sphere and cubic function model. The LTI system showed a linear mapping between the input and output signals, while time invariance indicated that the system will produce the same output signals if an input is used now or T seconds later, except for the time delay. The results showed that HPSO had better performance as compared to all other techniques that exist in the literature.

## 5. HPSO: Best-Fit Solution for Adaptive Filtering

It can be seen from the above experiments that the performance of HPSO was superlative as compared to other optimization techniques. HPSO is an effective approach for channel adaptive equalization, leveraging its global search capability to optimize equalizer coefficients and enhance the performance of communication systems by mitigating the effects of channel distortion and inter-symbol interference. This section will elaborate on the advantages, issues, and challenges faced by the HPSO in the optimization of adaptive filters.

### 5.1. Advantages of HPSO

#### 5.1.1. Exploiting Complementary Techniques

Hybrid PSO allows for the integration of different optimization algorithms or problem-solving methods that excel in different aspects. By combining their strengths, hybrid PSOs can overcome the limitations of individual algorithms and achieve better performance. For example, hybridizing PSO with genetic algorithms can leverage the exploration capabilities of both algorithms, leading to improved diversity and convergence toward optimal solutions.

#### 5.1.2. Enhanced Global and Local Search

Hybrid PSO combines the global search ability of PSO with the local search capabilities of other techniques. This integration allows for efficient exploration of the search space, enabling the algorithm to quickly identify promising regions and converge towards optimal solutions. The hybrid approach benefits from the balance between global exploration and local exploitation, providing better search efficiency.

#### 5.1.3. Adapting to Problem Characteristics

Different problems have distinct characteristics, such as multimodality, nonlinearity, or constraints. Hybrid PSO can be customized by selecting appropriate hybridization techniques based on the problem at hand. For instance, if a problem exhibits multimodality, combining PSO with niching techniques can enhance the algorithm’s ability to locate multiple optima. By adapting to the problem characteristics, hybrid PSO increases its effectiveness and robustness across diverse optimization scenarios.

#### 5.1.4. Handling Complex Constraints

Many real-world optimization problems involve complex constraints that must be satisfied. Hybrid PSOs can integrate constraint-handling techniques to ensure the feasibility of solutions. By incorporating constraint-handling mechanisms such as penalty functions, repair operators, or constraint satisfaction techniques, hybrid PSO can effectively handle constraints and generate feasible solutions, even in challenging constraint optimization problems.

#### 5.1.5. Domain-Specific Knowledge Incorporation

Hybrid PSO allows for the incorporation of problem-specific knowledge or heuristics. This customization leverages domain expertise to guide the search process toward more-promising regions of the search space. By integrating problem-specific knowledge, hybrid PSO can effectively exploit the problem structure and reduce the search space, leading to faster convergence and improved solution quality.

#### 5.1.6. Performance Versatility

Hybrid PSO’s flexibility enables it to adapt to various problem types and domains. It can be tailored to different optimization objectives, such as continuous optimization, discrete optimization, multi-objective optimization, or dynamic optimization. The ability to combine different algorithms and techniques makes hybrid PSO versatile, allowing it to tackle a wide range of optimization challenges effectively.

Hybrid particle swarm optimization (HPSO) stands out in dealing with complex optimization landscapes, which can be quite tricky due to the presence of multiple possible solutions and complex patterns. Due to its cooperative and adaptable nature, HPSO is particularly adept at exploring a wide range of potential solutions and skillfully adjusting its search strategy to navigate through intricate fitness landscapes. When faced with multi-objective optimization challenges, in scenarios where adaptive filtering requires finding a balance between conflicting objectives, such as fast convergence and precise tracking, HPSO’s strength lies in its ability to seamlessly integrate diverse optimization techniques. This integration enables HPSO to harmonize these contrasting objectives effectively. HPSO proves valuable in hybrid approaches, especially when the optimization task requires the integration of specific problem-solving strategies or domain expertise. This becomes particularly beneficial when traditional optimization methods struggle to handle complex problems due to their intricacy.

HPSO demonstrates adaptability in dynamic environments by dynamically adjusting parameters, incorporating updates based on local neighborhoods, and creating multiple swarms, allowing it to stay in sync with evolving optimization needs. This makes it a well-suited choice for scenarios where the underlying system’s characteristics change over time. In cases where substantial computational power is needed, HPSO can be parallelized across multiple processors or nodes, leading to faster optimization processes. This is particularly useful for tasks that require real-time processing capabilities. HPSO is highly versatile in handling various problem types and dynamic conditions with limited prior knowledge. Its hybrid nature strikes a perfect balance between exploring a broad range of solutions and refining them, making it particularly advantageous in adaptive filtering tasks.

### 5.2. Challenges and Limitations of HPSO

Hybrid PSO offers numerous advantages, as discussed earlier. However, like any optimization approach, it also faces certain challenges and limitations that should be taken into consideration.

#### 5.2.1. Algorithm Complexity

Hybrid PSO introduces additional complexity due to the integration of multiple optimization techniques or problem-solving methods. Managing the interactions and parameter settings between different components can be challenging. The design and implementation of a hybrid PSO algorithm require careful consideration to ensure effective cooperation and avoid conflicts between the integrated components.

#### 5.2.2. Hybridization Overhead

Integrating different optimization techniques or problem-solving methods in hybrid PSO may increase computational overhead. The hybridization process requires additional computational resources, such as memory and processing power. The impact on computational efficiency should be carefully assessed, especially when dealing with large-scale optimization problems or real-time applications.

#### 5.2.3. Algorithm Selection and Tuning

The success of hybrid PSO heavily depends on selecting appropriate optimization techniques or problem-solving methods to hybridize. Identifying the most-suitable algorithms or methods for a given problem can be challenging. Moreover, the tuning of parameters becomes more complex in hybrid PSO, as it involves optimizing the parameters of both the PSO algorithm and the integrated techniques. This parameter-tuning process requires expertise and extensive experimentation.

#### 5.2.4. Integration Compatibility

Integrating different optimization techniques or problem-solving methods in hybrid PSO might encounter compatibility issues. Some methods may require specific problem representations or assumptions that are not easily integrated with others. Ensuring compatibility and smooth integration of different components can be a challenge and may require adaptations or transformations to make them compatible.

#### 5.2.5. Increased Sensitivity to Problem Characteristics

Hybrid PSO’s performance can be sensitive to the problem characteristics and the choice of hybridization techniques. The effectiveness of hybrid PSO heavily relies on the compatibility and synergy between the integrated components and the problem at hand. In some cases, the hybrid approach may not provide significant improvements compared to standalone PSO or other individual techniques, particularly if the problem does not align well with the selected hybridization methods.

#### 5.2.6. Limited Generalizability

Hybrid PSO’s effectiveness may be problem-dependent, meaning that the success observed in one problem domain may not necessarily translate to other domains. The performance of a hybrid PSO is heavily influenced by the specific problem structure, objectives, and constraints. Consequently, the development of a hybrid PSO algorithm that performs well across diverse problem domains requires careful customization and adaptation to each specific problem.

#### 5.2.7. Increased Development and Maintenance Effort

Hybrid PSO requires additional effort in the development and maintenance stages. Combining multiple algorithms or methods necessitates expertise in those areas. As new optimization techniques emerge, the integration and evaluation of their compatibility with hybrid PSO may require continuous effort and expertise, making the development and maintenance of hybrid PSO algorithms more demanding.

## 6. Conclusions and Future Directions

### 6.1. Conclusions

Advancements in HPSO for adaptive equalization are pivotal for addressing limitations and enhancing practical implementation. Research must target simplifying the algorithm while upholding performance, optimizing hybridization for reduced computational overhead and automating parameter selection for enhanced efficiency. Efforts should prioritize enhancing integration compatibility, robustness, and generalizability across diverse equalization schemes and problem contexts. The utilization of user-friendly frameworks and libraries could streamline development and foster HPSO adoption, ultimately leading to improved BER performance in real-world adaptive equalization applications.

### 6.2. Future Directions

To tackle the algorithm complexity, one potential direction is to simplify HPSO. This involves analyzing the algorithm’s components and identifying areas where complexity can be reduced without compromising performance. Streamlining the algorithm can make it more accessible and easier to implement in practical scenarios, enabling wider adoption of HPSO for adaptive equalization.

Addressing hybridization overhead is another crucial future direction. Researchers can explore methods to optimize the integration of different optimization techniques in HPSO. This optimization can minimize computational overhead by intelligently determining when and how to employ local search mechanisms. By optimizing the hybridization process, the overall efficiency of HPSO can be improved, making it more suitable for real-time adaptive equalization applications.

Automated parameter selection is another promising direction to overcome the challenges associated with algorithm selection and tuning. By developing automated methods, such as metaheuristic optimization or machine learning algorithms, the task of selecting appropriate parameter values can be automated. This enables HPSO to adapt and optimize its parameters based on the specific adaptive equalization problem at hand, reducing the manual effort and subjectivity involved in parameter tuning.

Integration compatibility is a significant limitation that can be addressed through future research efforts. Investigating ways to enhance the compatibility of HPSO with different equalization schemes and systems is essential. Developing adaptive mechanisms that seamlessly integrate HPSO with diverse equalization techniques and architectures can significantly improve its effectiveness and versatility in adaptive equalization tasks. Measuring the stability of HPSO is another important avenue for future work. We intend to incorporate Monte Carlo to measure the stability of HPSO during the convergence process by obtaining the standard deviation and mean value of the MSE curve.

Enhancing the robustness and generalizability of HPSO is another crucial direction. Research can focus on reducing the algorithm’s sensitivity to problem characteristics and environmental conditions. By developing mechanisms to handle diverse channel conditions, noise levels, and signal variations, HPSO can become more reliable and applicable in real-world adaptive equalization scenarios. Efforts can be directed toward reducing the development effort required for HPSO implementation. This can involve the creation of user-friendly software frameworks, libraries, or toolkits that provide pre-defined implementations of HPSO for adaptive equalization. By simplifying the development process, researchers and practitioners can more readily adopt and utilize HPSO, accelerating its application and impact in the field of adaptive equalization.

## Figures and Tables

**Table 1:** Techniques used for adaptive equalization.

Technique	Limitations	Advantages
LMS	Susceptible to getting stuck in local optima [50].	Simplicity and ease of implementation [25]; Low computational complexity [26]; Achieves significant error reduction and convergence with reasonable computational resources.
RLS	High computational complexity and memory requirements [34]; Sensitive to numerical issues due to matrix inversion.	Fast convergence rate; Provides optimal filter updates [31]; Achieves improved convergence speed and provides accurate filter estimation.
PSO	May suffer from premature convergence and lack of diversity [51]; Requires fine-tuning of algorithm parameters.	Provides global search capability; Can handle complex and nonlinear optimization problems; Achieves optimal filter coefficients with enhanced convergence and improved equalization performance [38].
GA	Convergence speed may be slower compared to other algorithms; Requires a suitable representation of solutions and the design of appropriate genetic operators.	Can handle complex optimization problems; Provides a diverse set of solutions; Achieves improved equalization performance with diverse and globally optimal solutions [43].
Deep Learning	Requires a large amount of training data [52]; May suffer from overfitting.	Can adapt to complex and nonlinear channel characteristics; Provides high flexibility in modeling the equalization process [46]; Achieves superior equalization performance with accurate mapping of the input–output relationship.

**Table 2:** Advancements in the PSO algorithm.

Year	Advancement
1995	Introduction of PSO algorithm by Kennedy and Eberhart [1,82]
1997	Inclusion of inertia weight to balance exploration and exploitation [83]
1998	Exploration of PSO variants such as constriction factor approach [84]
1999	Incorporation of adaptive parameter settings for improved performance [85]
2001	Multi-objective PSO developed for handling optimization problems with multiple conflicting objectives [86]
2003	Hybridization of PSO with other metaheuristic or local search algorithms [87]
2004	Introduction of dynamic PSO variants to adapt to changing environments [88]
2006	Application of PSO in solving complex real-world problems, such as the optimization of neural networks and data clustering [89]
2008	Development of parallel and distributed PSO algorithms for enhanced computational efficiency [90]
2010	Integration of PSO with machine learning techniques for improved optimization and prediction tasks [91]
2012	Self-adaptive PSO algorithms introduced to dynamically adjust algorithm parameters during optimization [92]
2014	Improved PSO variants focusing on handling dynamic and uncertain environments [93]
2016	Application of PSO in feature selection, image processing, and bioinformatics problems [94]
2018	Exploration of hybrid PSO algorithms with deep learning models for enhanced optimization and decision-making [95,96]
2020	Advancements in multi-objective PSO algorithms for solving complex optimization problems with conflicting objectives [97]
2022	Development of PSO variants incorporating social-network-inspired behaviors for collective decision-making and coordination [98]

**Table 3:** PSO variants advantages and limitations for adaptive filtering.

Topic	Advantages	Limitations
Ring Topology	Local information sharing and cooperation [140] Promotes exploration and exploitation	Limited information propagation beyond immediate neighbors [99]
Dynamic Multi-Swarm	Efficient exploration of different regions Balances exploration and exploitation	Increased computational complexity [141] Requires careful tuning of swarm characteristics [107]
Fully Informed	Enhanced global exploration capability	Increased communication and computational overhead Sensitivity to parameter settings [112]
Dynamic Neighborhood	Adaptability to changing problem conditions [142] Improved exploration and exploitation	Complexity in managing dynamic neighbor relationships Additional computational overhead [114]
Hybridization	Leveraging strengths of multiple techniques Enhanced performance and solution quality	Increased algorithm complexity Requires expertise in multiple algorithms or methods [143]
Cooperative Particle Swarm	Knowledge exchange and cooperation among swarms Tackling large-scale optimization problems [144]	Increased communication and coordination requirements Complexity in swarm interaction management
Self-Organizing Hierarchical	Efficient exploration and coordination across different levels	Complexity in the hierarchical organization and coordination [145]
Comprehensive Learning	Adaptation to problem characteristics Improved convergence and solution quality	Requires problem-specific knowledge or heuristics [146] Increased algorithm complexity and parameter tuning effort

**Table 4:** Convergence of hybrid PSO with different values of *N*.

Number of Iterations	MSE (dB) *N* = 10	MSE (dB) *N* = 20	MSE (dB) *N* = 40	MSE (dB) *N* = 60
0	20	20	20	20
50	−12	−15	−28	−29
100	−20	−23	−29	−30
200	−21	−25	−29.5	−30
300	−19.5	−24.5	−29	−30.5
400	−20.5	−25	−29.5	−31
500	−22	−26	−30	−31

**Table 5:** BER performance of LMS and HPSO.

SNR	LMS	PSO VCF	HPSO
0	0.8922	0.8929	0.8929
2	0.8017	0.8019	0.8019
4	0.8402	0.8402	0.8402
6	0.8051	0.805	0.805131
8	0.74283	0.7428	0.7427
10	0.65015	0.6505	0.6505
12	0.5526	0.5527	0.5527
14	0.4026	0.4026	0.4026

**Table 6:** The 30D particles’ convergence comparison.

f(x)	fl			f2		
**Value**	**Mean**	**Iterations**	**Comp**	**Mean**	**Iterations**	**Comp**
PSO	7.1e−2	500	100%	55.44	500	100%
PSO-D	5.706e−53	500	100%	0	264	100%
PSO-DE	6.35e−20	412	70.3%	293	66.90%	
DMS	0.71 (2%)	500	100%	37.97	500	100%
DIMS-D	3.646e−54	500	100%	0	273	100%
DAIS-DE	3.85e−20	392	69.88%	0	320	66.48%
CL	1.056e−47	500	60%	0	312	60%
CL-D	3.486e−51	500	60%	0	279	60%
CEDE	7.19e−19	319	39.2%	0	258	37.63%
HP	1.0486e−5	50	80%	29.56	500	80%
HP-D	4.906e−111	500	80%	0	87	80%
HP-DE2	5.216e−15	55	3.99%	1.18e−13	227	12.80%

**Table 7:** The 60D particles’ convergence comparison.

f(x)	fl			f2		
**Value**	**Mean**	**Iterations**	**Comp**	**Mean**	**Iterations**	**Com**
PSO	x	x	x	150.12 (98%)	500	100%
PSO-D	1.266e−53	500	100%	0	273	100%
PSO-DE	1.46e−19	446	70.29%	0	291	66.94%
DMS	x	x	x	164.86 (62%)	500	100%
DMS-D	1.616e−54	500	100%	0.00%	269	100%
DMS-DE	9.58e−20	420	69.90%	0%	304	66.49%
CL	6.546e−44	500	60%	115%	500	60%
CL-D	52	500	60%	0.00%	275	60%
CEDE	1.42e−18	310	39.19%	0%	259	37.59%
HP	0.16	500	80%	6905%	500	80%
HP-D	6.566e−106	500	80%	0.00%	92	80%
HP-DE2	9.9e−15	3.98%	1.52e−228	228	12.78%	

**Table 8:** Mean function value of different PSO algorithms.

No.of Dimensions	PSO	DPSO	CPSO	HPSO
10	−185.37	−156.31	−300	−50.10100664
20	−81.951	−60.194	−296.087	−33.131
30	−48.781	−32.039	−281.737	−30.1019
40	−31.22	−19.417	−45.652	−27.6767
50	−18.537	−7.767	−3.9130	−26.4646
60	−18.537	−3.8835	−1.396	−26.2623
70	−13.659	−1.9418	−2.6628	−24.8481
80	−7.8049	−3.8835	−3.9138	−24.2424
90	−6.8293	0	−5.2173	−24.040
100	−4.878	0	0	−22.4242

**Table 9:** MSE performance for the linear channel.

SNR	LMS	PSO-CCF	PSO-VCF	HPSO
0	−0.0515	1.4433	5.051546	5.15464
50	−5.2062	−11.289	−13.9691	−18.0928
100	−8.6082	−11.753	−14.3299	−18.0928
150	−10.876	−11.907	−14.1237	−18.4021
200	−12.68	−11.598	−14.1237	−18.4536
250	−13.66	−11.804	−14.2268	−18.7113
300	−14.794	−11.804	−14.1237	−18.6598
350	−14.897	−11.959	−14.1237	−18.6598
400	−14.794	−11.753	−14.1753	−18.6598
450	−14.794	−11.753	−13.9691	−18.6082
500	−14.948	−11.959	−14.3814	−18.4021

**Table 10:** MSE performance for the nonlinear channel.

SNR	LMS	PSO-CCF	PSO-VCF	HPSO
0	−0.06568	4.9835	5.02463	12.2088
50	−2.159	−4.41707	−5.6075	−8.31691
100	−3.2676	−4.499	−5.8928	−8.6452
150	−3.6319	−4.622	−5.894	−8.6042
200	−3.9244	−4.4589	−5.8535	−8.6863
250	−4.1297	−4.4170	−5.93592	−8.6065
300	−4.70443	−4.41785	−5.85384	−8.6042
350	−4.8275	−4.2939	−5.89628	−8.76025
400	−4.78296	−4.2527	−5.77185	−8.604
450	−4.786296	−4.37602	−5.689	−8.8095
500	−4.745	−4.25287	−5.6077	−8.6863

## Data Availability

Not applicable.
